# Genetic variants of *BIN1*, *APOE* gene and GDF15 protein concentration in Alzheimer's disease patients

**DOI:** 10.1002/alz70856_104825

**Published:** 2026-01-07

**Authors:** Dominik Lewandowski, Mateusz Konieczny, Joanna Poszwa, Jolanta Florczak‐Wyspianska, Wojciech Owecki, Oliwia Szymanowicz, Artur Drużdż, Wojciech Kozubski, Jolanta Dorszewska

**Affiliations:** ^1^ Student Scientific Society of Poznan University of Medical Sciences, Poznań, Greater Poland, Poland; ^2^ Laboratory of Neurobiology, Department of Neurology, Poznań, Greater Poland, Poland; ^3^ Chair and Department of Neurology, Poznan Univeristy of Medical Sciences, Poznań, Greater Poland, Poland; ^4^ Department of Neurology, Municipial Hospital, Poznań, Greater Poland, Poland

## Abstract

**Background:**

Currently, Alzheimer's disease (AD) risk genetic variants include among others *APOE* and *BIN1*. *APOE* possesses 3 alleles: protective (ε2), neutral (ε3), and pathogenic (ε4). *APOE* gene is present in amyloid plaques and binds to Aβ peptides. *BIN1* gene is involved in the regulation of apoptosis and immune response. Growth and differentiation factor 15 (GDF15) is an inflammation‐associated hormone and, with diseases with inflammatory etiologies. GDF15 was shown to be associated with incident dementia including AD. Moreover, GDF15 is thought to be important in predicting dementia in both the long and short term. Individuals with higher levels of GDF15 are more likely to develop dementia. The role of GDF15 is poorly defined

**Method:**

The aim of the study was to analyze the *APOE* and *BIN1* genetic variants and GDF15 protein concentration in AD patients and related (CR), and unrelated (CU) controls with AD. The studies were conducted on 29 patients with AD. The control group included 25 CR and 27 CU. The *APOE* genotype was determined by real‐time PCR. The *BIN1* genotypes were determined by HRM and sequencing. GDF15 levels were analyzed by the ELISA method.

**Result:**

The study showed statistically significantly higher levels of GDF15 in CU compared to CR (*p* = 0.048) and similar levels in CR and AD. In CU carriers of *APOE* ε3ε3 and ε3ε4 (*p* = 0.0946) alleles trend towards higher GDF15 levels were demonstrated as compared with CR and AD. At the same time, CU with the *BIN1* CC genotype showed statistically significantly lower GDF15 levels compared to AD with *BIN1* CC (*p* = 0.050). The highest GDF15 levels were shown in individuals with the *BIN1* TT genotype, both CR and AD. In contrast to CU in CR and AD, only in *APOE* ε3ε3 individuals, the GDF15 protein level was 2‐3 times higher than the average score in the analyzed group.

**Conclusion:**

GDF15 appears to be more strongly involved in the development of dementia with immune regulatory factors (*BIN1* TT genotype).

